# Application of Causal Inference to Establish Assay Effect in the Absence of a Bridging Study: A Case Study of MenACWY‐CRM Conjugate Vaccine Data

**DOI:** 10.1002/pst.70069

**Published:** 2026-01-30

**Authors:** Meike Adani, Silvia Noirjean, Andrea Callegaro, Pavitra Keshavan, Marco Costantini

**Affiliations:** ^1^ GSK Siena Italy; ^2^ GSK Rixensart Belgium

**Keywords:** assay, bridging study, inverse probability weighting, MenACWY‐CRM, overlap weighting, propensity score

## Abstract

During a vaccine development program, if the assay used to measure immunological endpoints is changed, ideally, a bridging study is performed to establish the relationship between results obtained with the new and previous assay. However, this is not always feasible, and when bridging study data are absent, this can limit the ability to use historical study information to strengthen evidence generated in the clinical program. We present a case study on GSK's quadrivalent meningococcal vaccine (MenACWY‐CRM), where the immunogenicity assay was changed over time. A large amount of study data was collected in randomized controlled clinical trials, providing a valuable source of information to support vaccine development, but the introduction of the new assay complicated the comparison of antibody responses across studies. Several causal inference techniques, developed for the analysis of non‐randomized studies, can be used to estimate the assay bridging effect and, as observed in our case study, address the presence of confounding factors resulting from pooling group data from different sources. Cutting‐edge propensity score‐based methods were evaluated, highlighting their advantages and limitations. Within the family of propensity score weighting methods, the widely used inverse probability weighting was compared to the novel overlap weighting technique. The latter was shown to resolve the problem of extreme weights in a situation where there was poor overlap in covariate distribution between two groups. Automated selection of specific methods should be approached with caution, carefully considering the different estimands targeted by different methods.

## 1 Introduction

The quadrivalent meningococcal glycoconjugate vaccine, MenACWY‐CRM (*Menveo*, GSK), that uses non‐toxic diphtheria cross‐reacting material 197 (CRM_197_) as carrier protein, induces active immunization against invasive meningococcal disease caused by *Neisseria meningitidis* serogroups A, C, W, and Y [1]. MenACWY‐CRM is currently approved in over 60 countries, with more than 82 million doses distributed worldwide since 2010 (GSK data).

Studies conducted in different age groups and various countries supported the approval of the original MenACWY‐CRM lyo/liquid formulation, composed of a lyophilized serogroup A component and liquid serogroups C, W, and Y component [2]. For the licensing of a new fully liquid formulation (*Menveo Liquid*, GSK), two clinical studies (NCT03652610, NCT03433482) conducted in 2018–2019 evaluated non‐inferiority to the licensed MenACWY‐CRM presentation [3, 4]. Non‐inferiority was demonstrated for serogroup A, which was changed to a liquid composition.

During the clinical development of the fully liquid presentation, the assay used to measure immune responses was changed to improve efficiency of the testing procedure and because of new regulatory requirements. Studies conducted on the fully liquid formulation adopted the agar overlay assay for measuring human serum bactericidal antibody (hSBA) titers against serogroups A, C, W, and Y [3, 4], while previous studies used the manual tilt assay [5, 6, 7, 8, 9, 10]. The serogroup A strain also differed between studies: the 3125 strain was used in studies of the fully liquid formulation that used the new assay, while the F238 strain was used in studies that used the manual tilt assay [11]. The new assay was also used in a recent study in which MenACWY‐CRM was used as control [12]. No formal bridging study was conducted to determine if the assay change had any effect on measured immunological responses to MenACWY‐CRM. Estimation of the assay effect would enable fair comparisons of antibody responses across studies and allow the use of historical study data to strengthen evidence generated in the clinical program.

The aim of this case study was to evaluate the assay bridging effect due to the change from manual tilt to agar overlay assay; for serogroup A, this was the combined effect of a different assay and strain. This was estimated by using pooled data from MenACWY‐CRM studies, where one of the two assays was used to measure hSBA titers against serogroups A, C, W, and Y. Only the MenACWY‐CRM lyo/liquid formulation was considered since the fully liquid formulation was only tested with the new agar overlay assay. Immunological responses for the group that received MenACWY‐CRM and whose hSBA titers were measured with the agar overlay assay were compared against the group that received the same vaccine but whose hSBA titers were measured with manual tilt. Since these two groups of participants were created by pooling data from multiple studies, they showed a partially different distribution in some baseline characteristics (e.g., demographics), which could affect the hSBA titers, that is, they could be confounders. Causal inference approaches, specifically propensity score‐based methods, developed for the analysis of non‐randomized trials [13, 14], were therefore explored with the aim of controlling for these potential confounding factors and thus disentangling an average assay effect.

## 2 Methods

### 2.1 MenACWY‐CRM Studies and Analysis Sets

Data were pooled from eight studies in which participants received MenACWY‐CRM as primary or control vaccine. The agar overlay assay was used to measure hSBA titers in three recent studies in which MenACWY‐CRM was given as control: two studies of the fully liquid formulation [3, 4] and the QUINTET study [12]. Five older studies used the manual tilt assay to measure hSBA titers, of which two were pivotal studies for the regulatory approval of MenACWY‐CRM [5, 6, 9] and three were MenACWY‐CRM studies that were selected to address the heterogeneity of the countries involved [7, 8, 10]. Precision of both assays used to measure hSBA titers in the clinical studies was validated within predefined acceptance criteria during clinical assay development.

The analysis dataset was restricted to age groups and countries for which immunogenicity data were available for both assays, which included participants aged 10–40 years from Italy, Canada, Russia, and the United States (US), and participants with evaluable hSBA titers at 1 month post‐vaccination for at least one of the serogroups (A, C, W, Y). Analysis results are shown separately for serogroup A, for which the assay effect was due to the combination of assay change and different strain, and remaining serogroups C, W, and Y.

**FIGURE 1:**
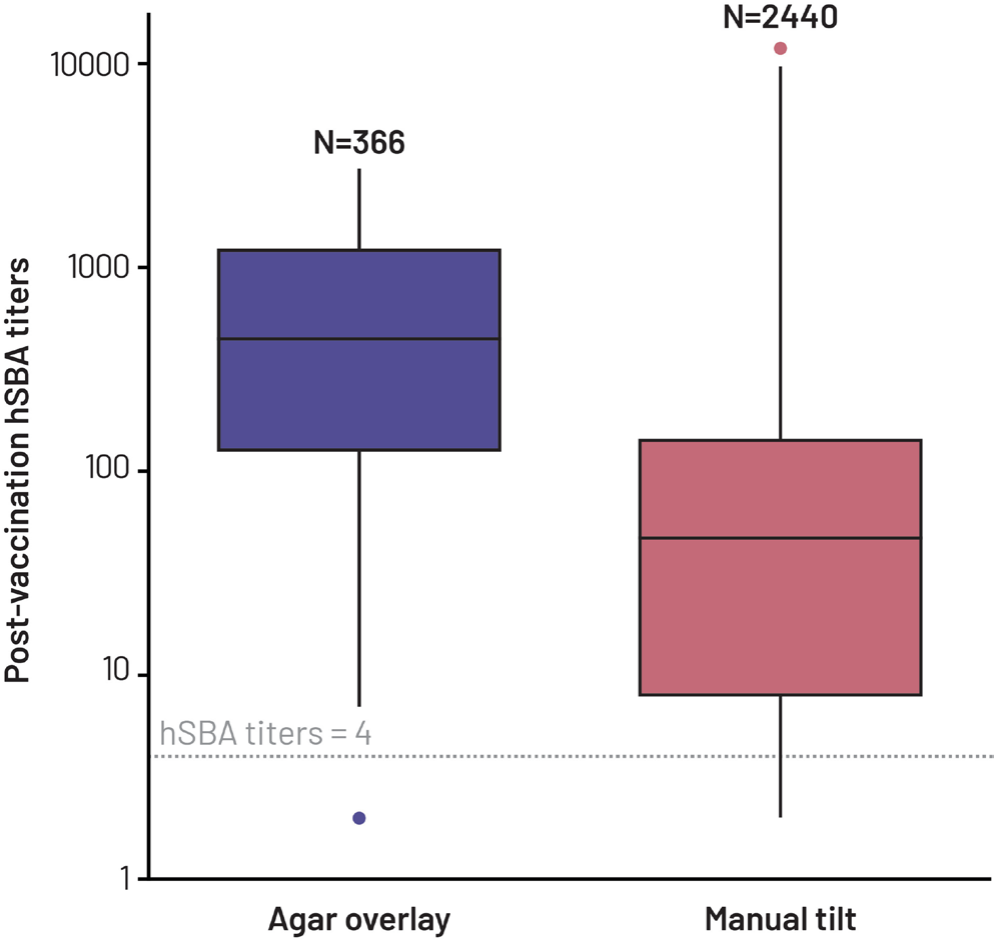
Distribution of post‐vaccination hSBA titers against serogroup A in the Agar Overlay group and the Manual Tilt group. Box plot showing median (horizontal lines), 25th and 75th percentiles (boxes), maximum and minimum values (vertical bars), and outliers (dots). hSBA, human serum bactericidal antibody; *N*, number of participants in group.

The main analyses presented are for serogroup A. These used data from 2806 participants, of whom 366 were from studies using the agar overlay assay (Agar Overlay group) and 2440 from studies using the manual tilt assay (Manual Tilt group). The demographic characteristics of the two groups are shown in Table 1, while Figure 1 shows the distribution of observed post‐vaccination hSBA titers for the two groups. The observed response for the Agar Overlay group was generally higher than that for the Manual Tilt group. Since the data were pooled from different studies, the distribution of certain characteristics differed between the two groups, particularly age group and country of origin. Specifically, most participants (77.0%) in the Agar Overlay group were in the 18–40 years age group, while in the Manual Tilt group, most participants (73.1%) were in the 10–17 years age group, and most participants (79.1%) in the Manual Tilt group resided in the United States, while 34.7% and 35.8% of participants in the Agar Overlay group were from Canada and Russia, respectively. There were also partial imbalances in the distribution of race. Such an imbalance in covariates distribution among the two groups required a careful evaluation of causal inference methodologies to control for confounding factors in a specific situation where poor overlap was observed for some of the covariates. Propensity score weighting methods were therefore leveraged to achieve covariate balance and estimate a causal effect of the assay change.

**TABLE 1:** Demographic characteristics of participants from eight pooled MenACWY‐CRM studies included in the analyses on meningococcal serogroup A.

Characteristic	Agar Overlay group (*N* = 366)	Manual Tilt group (*N* = 2440)	Total (*N* = 2806)
Age group, *n* (%)			
10–17 years	84 (23.0)	1783 (73.1)	1867 (66.5)
18–40 years	282 (77.0)	657 (26.9)	939 (33.5)
Sex, *n* (%)			
Female	211 (57.7)	1269 (52.0)	1480 (52.7)
Male	155 (42.3)	1171 (48.0)	1326 (47.3)
Race, *n* (%)			
White	329 (89.9)	2111 (86.5)	2440 (87.0)
Black/African American	12 (3.3)	184 (7.5)	196 (7.0)
Asian	19 (5.2)	53 (2.2)	72 (2.6)
Other	6 (1.6)	92 (3.8)	98 (3.5)
Country, *n* (%)			
Italy	70 (19.1)	367 (15.0)	437 (15.6)
Canada	127 (34.7)	33 (1.4)	160 (5.7)
United States	38 (10.4)	1929 (79.1)	1967 (70.1)
Russia	131 (35.8)	111 (4.5)	242 (8.6)
Baseline serostatus for serogroup A,^a^ *n* (%)			
Seronegative	303 (82.8)	2295 (94.1)	2598 (92.6)
Seropositive	42 (11.5)	126 (5.2)	168 (6.0)
Unknown	21 (5.7)	19 (0.8)	40 (1.4)

*Note:* Agar Overlay group, group for which immunogenicity was assessed by agar overlay assay; Manual Tilt group, group for which immunogenicity was assessed by manual tilt assay; *N*, number of participants in group; *n*, number of participants in category.

^a^ Seronegative: participants with pre‐vaccination human serum bactericidal antibody (hSBA) titers < 4; seropositive: participants with pre‐vaccination hSBA titers ≥ 4.

### 2.2 Estimands

The following notation is used throughout this work, with the assay $A_{i}$ defining the two groups to be compared. For each participant 𝑖,

- $A_{i}$: assay group (1 = Agar Overlay group; 0 = Manual Tilt group).
- $X_{i}$: vector of baseline covariates (including age group, sex, race, and country, as defined in Table 1).
- $Y_{i}$: outcome (log10 hSBA titers 1 month after vaccination; the log10 transformation of the hSBA titers was used in order to work with approximately normally‐distributed outcome data, as commonly done in vaccine studies).

According to the potential outcome framework that was adopted for defining the causal effect of interest [15, 16], it is possible to define $Y_{i}\left(a\right)$ as the potential outcome of participant i if the assay used to measure their outcome were $a\left(a\in \left\{0,1\right\}\right)$. In other words, $Y_{i}\left(1\right)$ is the outcome that *would be* observed with the agar overlay assay, and $Y_{i}\left(0\right)$ is the outcome that *would be* observed with the manual tilt assay. This definition is based on the Stable Unit Treatment Value Assumption, SUTVA [17, 18].

Assumption 1 (SUTVA) There is (i) no interference between trial participants and (ii) no different versions of the same treatment.

In the MenACWY‐CRM case study, the treatment to which Assumption 1 (ii) refers is the assay used to measure hSBA titers (agar overlay or manual tilt). Thus, the assumption states that the potential outcome for one participant (i.e., hSBA titers measured with agar overlay or manual tilt assay) does not depend on the assay used to measure hSBA titers for other participants. Additionally, it states that there are no hidden variations of the agar overlay or manual tilt assay, which is a reasonable assumption to make because the potential variability of the assays over time is monitored during assay life cycle management and controlled within a prespecified acceptance range.

Under SUTVA, one of the two potential outcomes, $\left\{Y_{i}\left(0\right),Y_{i}\left(1\right)\right\}$, can be observed: specifically, $Y_{i}\left(A_{i}\right)=Y_{i}$ for $A_{i}\in \left\{0,1\right\}$. On the other hand, the counterfactual outcome, $Y_{i}\left(1-A_{i}\right)$, which is the outcome that would be observed with the assay that was not used, is missing for every participant i.

A causal effect is defined as a comparison of potential outcomes on a common set of units. Here, the interest is the average assay effect, which can be formally defined as follows:

(1) $$\tau =E_{g}\left[Y_{i}\left(1\right)-Y_{i}\left(0\right)\right]$$

where $E_{g}\left[\cdot \right]$ is the expected value over a target population g. In other words, the average assay effect in the target population is defined as the expected difference between the log10 hSBA titers measured with the agar overlay and manual tilt assay. A positive difference meant a higher immune response obtained using the agar overlay as compared to manual tilt. Results were then back‐transformed to estimate the geometric mean ratio (GMR) of hSBA titers in the Agar Overlay group versus Manual Tilt group ($\mathrm{GMR}=10^{\tau }$). Corresponding 95% confidence intervals (CIs) were computed and also back‐transformed to obtain 95% CIs for the GMR [19].

### 2.3 Identification of Causal Effect

To identify the causal effect defined in Equation (1) from the observed data, unconfoundedness and positivity assumptions were needed.

Assumption 2 (Unconfoundedness) $A_{i}⊥\left\{Y_{i}\left(0\right),Y_{i}\left(1\right)\right\}\;|\;X_{i}$.

Unconfoundedness, also known as conditional exchangeability, is the assumption that there are no unmeasured confounders of the assay effect on hSBA titers when conditioning on the observed covariates [18, 20]. In other words, the vector $X_{i}$ includes all potential measured confounders.

In the absence of randomization, there is no guarantee that the conditional exchangeability assumption holds, due to the risk of residual unmeasured confounders [21]. However, even if this is generally not testable, the unconfoundedness assumption is required to draw causal conclusions from non‐randomized groups.

Assumption 3 (Positivity)

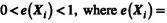
 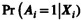

Positivity, also known as the overlap assumption, is the assumption that, for each participant i, the conditional probability of having the hSBA titers measured with the agar overlay assay, given the observed covariates, is bounded between 0 and 1. This conditional probability, also called the “propensity score,” [20] is denoted with $e\left(X_{i}\right)$ throughout the manuscript.

Plausibility of the positivity assumption was assessed in the selection of the studies, ensuring that all covariates were represented in the two different groups.

### 2.4 Estimation of Propensity Score

In our case study, the propensity score was unknown and needed to be estimated. We used a logistic model of the form $\text{logit}\;e\left(X_{i}\right)=X_{i}^{\prime }\beta$, where the first element of $X_{i}$ is 1 so that the model includes an intercept. A fixed‐effect model was considered without study‐specific random effects that would cause convergence issues since only one of the two assays was used within each study. Additionally, no study‐level confounders were anticipated and estimating the propensity score with a fixed‐effect logistic regression model ensures exact balance property, which is not guaranteed if other models are used [18, 22].

The estimated propensity score for the *i*th participant was denoted with $\hat{e}\left(X_{i}\right)$. Once estimated, the propensity score was used to define the weights for the different estimation methods, each of which corresponded to a specific target population g and causal estimand [15]. An unbiased estimator for the average assay effect was given by the weighted difference of the outcome between the two assay groups (Hàjek estimator): [18, 19]

$$\hat{\tau }=\frac{\sum_{i}w_{i}A_{i}\;Y_{i}}{\sum_{i}w_{i}A_{i}}-\frac{\sum_{i}w_{i}\left(1-A_{i}\right)Y_{i}}{\sum_{i}w_{i}\left(1-A_{i}\right)}$$

where $w_{i}$ represent the weights. From the general class of propensity score weighting methods, two methods were applied and compared in this case study: inverse probability weighting (IPW) and overlap weighting (OW) [18, 22]. With IPW, weights were defined as $w_{i}=1/\hat{e}\left(X_{i}\right)$ for units in the Agar Overlay group $\left(A_{i}=1\right)$ and $w_{i}=\frac{1}{1-\hat{e}\left(X_{i}\right)}$ for units in the Manual Tilt group $\left(A_{i}=0\right)$. Each unit was therefore weighted by the inverse of the probability of being assigned to its actual group. The target population for IPW was the combination of units in the two assay groups in equal proportion to their representation in the sample, and the causal estimand was the average assay effect of the total sample of the two groups combined. With OW, weights were defined as $w_{i}=1-\hat{e}\left(X_{i}\right)$ for units in the Agar Overlay group $\left(A_{i}=1\right)$ and $w_{i}=\hat{e}\left(X_{i}\right)$ for units in the Manual Tilt group $\left(A_{i}=0\right)$. Each unit was weighted by its probability of being assigned to the opposite group. The target population for OW was the set of units whose characteristics could appear with substantial probability in either assay group (having the most overlap), and the causal estimand was the average assay effect on the overlap population.

The performance of the different propensity score methods has already been evaluated in the literature under different simulation scenarios, with decreasing overlap in the covariates distribution. Even if in this case study we aimed to estimate an assay effect rather than a treatment effect, the same considerations on model performance apply. Therefore, OW, which consistently showed unbiased treatment effect estimates with lower bias in cases of poor overlap, was compared to the more widely used IPW in this case study [18, 22].

In the primary analysis, baseline log10 hSBA titers were not included as a covariate in the model of the propensity score to avoid bias resulting from the measurement of hSBA titers at baseline with two different assays. Sensitivity analyses were conducted to estimate the average assay effect when baseline serostatus was included in the propensity score model, with a separate analysis conducted for baseline seronegative participants. Baseline serostatus was dichotomized as seronegative for participants with pre‐vaccination hSBA titers < 4 and seropositive for participants with pre‐vaccination hSBA titers ≥ 4. As suggested by Goldschneider et al. [23, 24], participants with values below this cut‐off are likely to be more susceptible to infection, being at higher risk of systemic disease from meningococcal strains. Considering that this condition would not change based on the assay used, and that post‐vaccination immune responses for seronegative participants would not be influenced by different baseline hSBA titers, sensitivity analyses were carried out on that population only to assess the robustness of the primary results.

All analyses were performed in R; the PSweight package was leveraged for implementation of the propensity score weighting methods [19].

## 3 Results

### 3.1 Propensity Score

**FIGURE 2:**
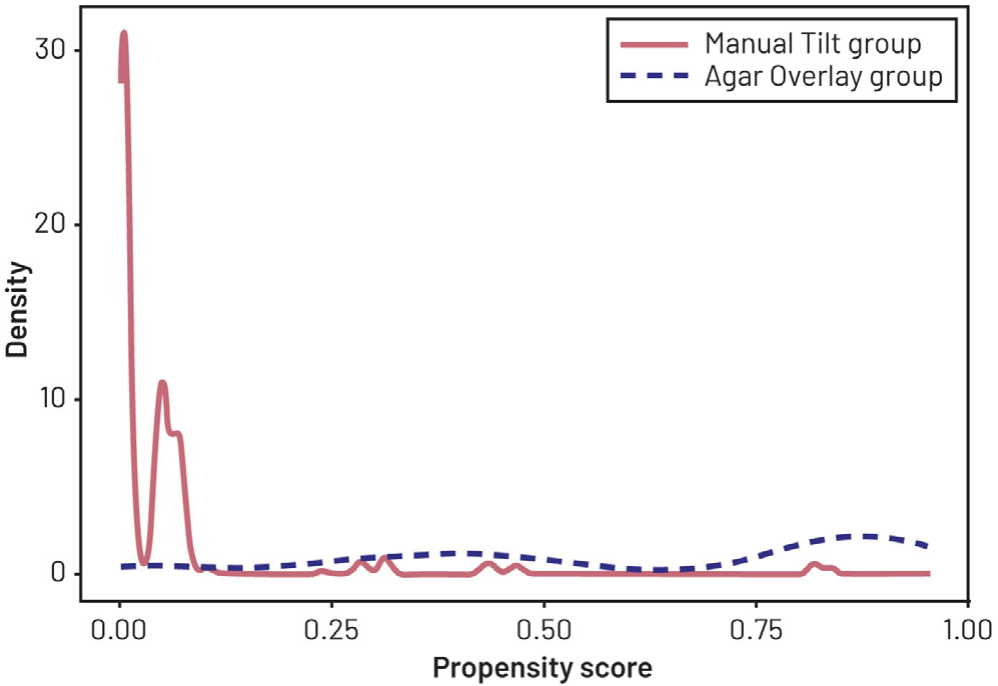
Estimated propensity score distribution in the Agar Overlay group and the Manual Tilt group. Density: Probability density function.

**FIGURE 3:**
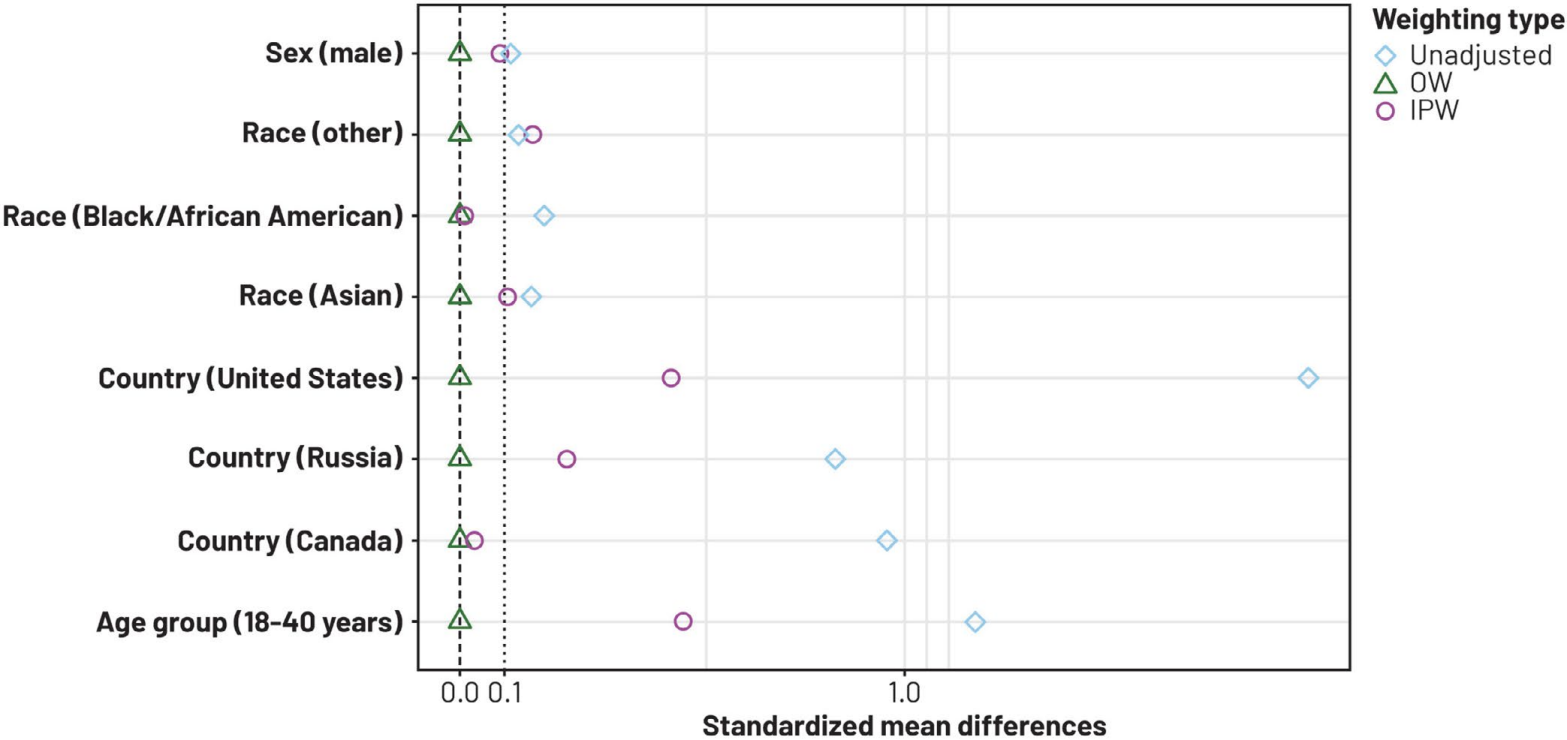
Balance of covariates with different propensity score weighting methods. OW, overlap weighting; IPW, inverse probability weighting.

The distributions of the estimated propensity score by assay group, as shown in Figure 2, demonstrate poor overlap in the tails, due to differences in the marginal distributions of the covariates between groups. Figure 3 shows the standardized mean differences, or absolute standardized differences (ASD) [19], of all covariates included in the model for the propensity score, before and after weighting. Specifically, for each of the *k*th covariate $X_{k}$, the ASD is calculated as the absolute weighted mean difference between assay groups scaled by the square root of the pooled within‐group variance:

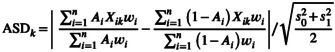

where $w_{i}$ is the weight for unit 𝑖, and $s_{1}^{2},$ $s_{0}^{2}$ are unweighted sample variance of $X_{k}$ in the Agar Overlay and the Manual Tilt groups [19, 22]. An ASD greater than 0.1 denotes an imbalance in the marginal distribution of the covariate by assay group [22]. It is evident that both weighting methods reduced the differences between groups, with standardized mean differences being exactly zero with OW, indicating exact balance for all covariates.

### 3.2 Average Assay Effect for Serogroup A

The combined effect of using a different assay and strain was analyzed for serogroup A, based on the differences between log‐transformed hSBA titers of the Agar Overlay group versus Manual Tilt group. Differences were back‐transformed to obtain the estimated GMR of hSBA titers against serogroup A for the target population.

The limited amount of data with values near to the assay cut‐off did not allow reliable estimation of GMR in the lower part of the range only, which could be different from the overall expected value. Therefore, the average causal effect of the assay, defined in Equation (1) as the expected value for the difference of two potential outcomes, was estimated considering the entire range of the assay.

**FIGURE 4:**
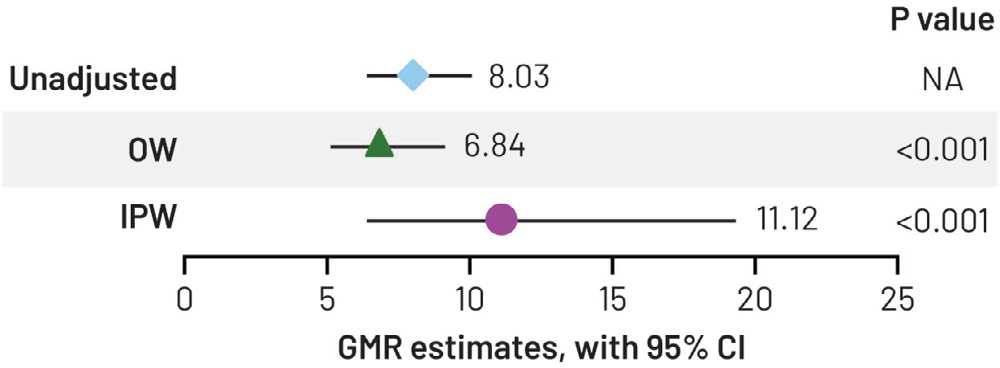
Average assay effect, as shown by geometric mean ratio (GMR) of hSBA titers against serogroup A in the Agar Overlay group versus the Manual Tilt group with inverse probability weighting (IPW), overlap weighting (OW), and no weighting (unadjusted). CI, confidence interval; NA, not applicable.

The estimated GMR was 11.12 (95% CI [6.39; 19.34]; *p* < 0.001) with IPW and 6.84 (95% CI [5.12; 9.14]; *p* < 0.001) with OW (Figure 4). Propensity score values near to 0 or 1 led to extreme weights when using IPW, resulting in a wide CI and consequently a much higher level of uncertainty with IPW than with OW.

Since weighted samples generally lead to a lower precision than the corresponding unweighted samples, the effective sample size (ESS) after IPW and OW was computed. ESS is a metric that measures how weighting increases uncertainty in estimates; specifically, it represents the size of an unweighted sample that would achieve approximately the same precision as the weighted sample estimate. It is calculated as the square of the summed weights divided by the sum of squared weights [25]. In our case study, estimated ESS after IPW and OW was, respectively, 68 and 584, confirming a higher precision when using OW.

As described in Section 2.4, sensitivity analyses were conducted to include baseline serostatus as a covariate in the propensity score model. Within this scope, OW was used to test the hypothesis that baseline serostatus was not influenced by the assay. This gave an average assay effect of 1.05 (95% CI [0.93; 1.18]; *p* = 0.45). Since the average assay effect on baseline serostatus was not significant (*p* > 0.05), this was included as a covariate in the propensity score model used in OW. This showed an average assay effect (Table 2) that was consistent with the primary analysis result. When the same analysis was performed using data from seronegative participants only (Table 2), again, the result was consistent with the primary analysis result.

**TABLE 2:** Sensitivity analyses of average assay effect when baseline serostatus was included in the propensity score model with overlap weighting, as shown by geometric mean ratio (GMR) of hSBA titers against serogroup A in the Agar Overlay group versus the Manual Tilt group.

Method	GMR estimate	95% CI	*p*
Baseline serostatus included	6.21	4.62, 8.35	< 0.001
Baseline seronegative participants included^a^	6.11	4.52, 8.25	< 0.001

Abbreviations: CI, confidence interval; hSBA, human serum bactericidal antibody.

^a^ Participants with pre‐vaccination human serum bactericidal antibody titers < 4.

### 3.3 Average Assay Effect for Serogroups C, W, and Y

Estimation of the average assay effect for the other three serogroups showed that, for serogroups C and W, the average assay effect on the target population was not significant with either IPW (GMR 2.91 [0.91, 9.32] for serogroup C, 1.29 [0.54, 3.07] for serogroup W) or OW (GMR 1.11 [0.70, 1.75], 0.72 [0.50, 1.02], respectively) (Table 3). For serogroup Y, the average assay effect was significant with both weighting methods (GMR 3.29 [1.37, 7.88] with IPW, 1.73 [1.21, 2.47] with OW) (Table 3). Due to the extreme weights of IPW for all three serogroups, again, the level of uncertainty was higher with IPW than with OW.

**TABLE 3:** Average assay effect, as shown by geometric mean ratio (GMR) of hSBA titers against serogroups C, W, and Y in the Agar Overlay group versus the Manual Tilt group with inverse probability weighting and overlap weighting.

Serogroup weighting method	GMR estimate	95% CI	*p*
Serogroup C			
Inverse probability weighting	2.91	0.91, 9.32	0.073
Overlap weighting	1.11	0.70, 1.75	0.668
Serogroup W			
Inverse probability weighting	1.29	0.54, 3.07	0.561
Overlap weighting	0.72	0.50, 1.02	0.061
Serogroup Y			
Inverse probability weighting	3.29	1.37, 7.88	0.008
Overlap weighting	1.73	1.21, 2.47	0.002

Abbreviations: CI, confidence interval; hSBA, human serum bactericidal antibody.

## 4 Discussion

We leveraged causal inference methodologies to estimate the effect of changing a clinical study assay in the absence of bridging study results. Specifically, this case study examined the effect of using a new agar overlay assay versus a previously used manual tilt assay to generate antibody response data following MenACWY‐CRM vaccination. Ideally, formal assay bridging studies are carried out on the same samples to compare measurements within the same runs, thereby controlling sample and run variability. However, sample and run variability were present in both assay groups in the analysis and were additionally controlled for during the clinical assay maintenance phase, ensuring minimal impact on the estimation. The causal inference methodologies used in this case study were therefore useful in providing an estimation of the average assay effect for the immune responses of trial participants.

An Agar Overlay group and Manual Tilt group were generated by pooling data from eight studies in which participants received MenACWY‐CRM as the primary vaccine [5, 6, 7, 8, 9, 10] or control [3, 4, 12]. In the pooled dataset, there were imbalances in demographic characteristics between the two groups and it was essential to take these differences into account to estimate a causal effect of the assay change. This was accomplished by using propensity score‐based methods (propensity score weighting). The two weighting methods (IPW and OW) reduced group differences for the observed covariates, with OW leading to exact balances. The exact balancing property of OW has been demonstrated theoretically [18] and our finding supports its use where comparator groups are very different. IPW is widely used but has limitations when propensity score values approach 0 and 1, where weights become extreme; it is well known that this method exhibits high variance due to causal comparisons that are highly uncertain [22, 26]. In contrast, overlap weights are always bounded between 0 and 1, addressing the situation of extreme weights [22].

In the analysis of hSBA titers against serogroup A after MenACWY‐CRM vaccination, we found that the assay bridging effect was highly variable with application of IPW due to extreme weights. With OW, even for propensity score values that were near to 0 or 1, extreme weights were not obtained, thereby overcoming the limitations of IPW. The same behavior was observed in the serogroups C, W, and Y analyses, which showed that OW was preferable to IPW for obtaining stable estimates for the assay bridging effect.

Participants with extreme propensity scores are typically down‐weighted with OW and have a reduced influence on the results. For this reason, it should be noted that OW changes the target population from the overall population represented by the sample (as targeted by IPW) to the “overlap” population [22]. The way the population is changed depends on the weights and sample features that are not evident in advance. Therefore, in practice, it is important to describe the resulting target population for the analysis, in terms of baseline characteristics of the weighted pseudopopulation [22]. Table 4 shows the baseline demographic characteristics for the pseudopopulation (overlap population, using OW) in our case study, which are exactly balanced between the two assay groups. Comparing against proportions for the original unweighted population, a slightly different distribution was observed, especially for covariates “age group” and “country,” which were the most unbalanced in the original sample.

**TABLE 4:** Demographic characteristics for participants in the weighted pseudopopulation (overlap population, using overlap weighting) and unweighted population. For each variable, weighted proportions were computed using overlap weights with the following formula: $\frac{\sum_{i}w_{i}A_{i}X_{i}}{\sum_{i}w_{i}A_{i}}$, where $X_{i}$ represents the covariate of participant i and $A_{i}$ is the assay group indicator (1 = Agar Overlay group; 0 = Manual Tilt group).

Characteristic	Overlap population (%)	Unweighted population (%)
Age group		
10–17 years	45.4	66.5
18–40 years	54.6	33.5
Sex		
Female	53.6	52.7
Male	46.4	47.3
Race		
White	91.8	87.0
Black/African American	4.3	7.0
Asian	2.4	2.6
Other	1.5	3.5
Country		
Italy	26.8	15.6
Canada	9.7	5.7
United States	25.8	70.1
Russia	37.8	8.6

One of the main limitations of propensity score‐based methods is that they rely on several assumptions. In this case, the assay bridging effect was estimated on populations for which immunogenicity data were available for both assays, which consisted of participants from a restricted set of countries and age groups. Additionally, no subgroup analyses were performed to explore potentially different assay effects based on different covariate distributions due to the limited sample size of subgroups with overlapping covariates. With IPW and OW, we estimated a marginal effect that considers the entire distribution of the included covariates.

Also, the possibility of model misspecification or the presence of unmeasured confounders was not evaluated. Additionally, the “traditional regression” method adjusted for all covariates was not considered; propensity score methods have theoretical advantages over the conventional method, which is an unbiased estimator of the average treatment effect if the model is not misspecified (e.g., no treatment heterogeneity; see, e.g., Shi et al. [27]) and can give lower precision when groups differ greatly in observed characteristics [28]. Finally, Bayesian causal inference methods were not included in the evaluation; a traditional frequentist framework was rather considered due to the limited a priori knowledge that could meaningfully inform a Bayesian framework. However, the Bayesian approach to inference is frequently employed in causal inference literature and its use could be beneficial where informative prior distributions can be specified [29].

In conclusion, where two non‐randomized groups are to be compared, participant characteristics are often quite different, leading to extreme tails in the propensity score distribution. We showed through a real‐life case study that OW can address this problem, providing estimates with smaller variance than the widely used IPW. The results on the estimated average assay effect for MenACWY‐CRM should be considered for future comparisons where new data are generated with the agar overlay assay. Our findings should also help raise awareness of the implications of using historical data to perform comparisons or inform current decision‐making. Finally, this work shows the potential of causal inference methodologies in overcoming the absence of a bridging study, while carefully considering the strengths and limitations of the different methods and corresponding estimands.

## Author Contributions

All authors participated in the design or implementation or analysis, and interpretation of the study; and the development of this manuscript. All authors had full access to the data and gave final approval before submission. Menveo and Menveo Liquid are trademarks owned by or licensed to the GSK group of companies.

## Funding

GSK funded this research and was involved in all stages of research conduct, including analysis of the data. GSK also took charge of all costs associated with the development and publication of this manuscript.

## Conflicts of Interest

Meike Adani is employed by GSK. Silvia Noirjean, Andrea Callegaro, Pavitra Keshavan, and Marco Costantini are employed by GSK and hold financial equities in GSK. The authors declare no other financial and non‐financial relationships and activities.

## Data Availability

Anonymized individual participant data and study documents can be requested for further research from www.clinicalstudydatarequest.com.
